# Comparison of IV Nalbuphine versus ibuprofen for Postoperative Pain control in cesarean section

**DOI:** 10.12669/pjms.41.9.11016

**Published:** 2025-09

**Authors:** Mehnaz Khakwani, Rashida Parveen, Hajra Sultana

**Affiliations:** 1 Mehnaz Khakwani, FCPS Department of Obstetrics and Gynecology, Nishtar Medical University, Multan, Pakistan; 2 Dr. Rashida Parveen, FCPS, MRCOG. Associate Professor Gynae, Nishtar Medical University, Multan, Pakistan; 3 Hajra Sultana, FCPS Department of Obstetrics and Gynecology, Nishtar Medical University, Multan, Pakistan

**Keywords:** Cesarean section, Drowsiness, Ibuprofen, Nalbuphine, Nausea

## Abstract

**Objective:**

To evaluate the effectiveness of intravenous (IV) nalbuphine versus IV ibuprofen for postoperative pain relief following cesarean section under general anaesthesia.

**Methodology:**

This randomized controlled trial was conducted at the department of obstetrics and gynecology, Nishtar Hospital, Multan, Pakistan from March 2024 to August 2024. A total of 80 females, aged between 18-45 years, gestational age above 32 weeks, body mass index (BMI) ≤ 30 kg/m^2^, who were planned to undergo elective cesarean were included. Post-operatively, females in Ibuprofen group were given 800 mg Ibuprofen in 200 ml normal saline intravenously (IV) at the time of arrival in the ward. Women in Nalbuphine group received 20 mg nalbuphine in 200 ml normal saline IV at the time of arrival in the ward. Post-operative visual analog scale (VAS) scores, need for rescue analgesia, satisfaction, and side effects were noted.

**Results:**

Post-operatively, the Nalbuphine group showed better pain control than the Ibuprofen group at one hour (p<0.001), three hours (p<0.001), and six hours (p=0.011). Rescue analgesia was required more frequently in the Ibuprofen group (70.0% vs. 17.5%, p<0.001). Patient satisfaction was higher in the Nalbuphine group (62.5% vs. 12.5%, p<0.001). Nausea (20.0% vs. 10.0%, p=0.210), and gastrointestinal discomfort (2.5% vs. 12.5%, p=0.090) were statistically similar. Drowsiness was more frequent with Nalbuphine group (25.0% vs. 5.0%, p=0.012).

**Conclusion:**

This study demonstrated that IV Nalbuphine was more effective than IV Ibuprofen for postoperative pain relief following cesarean section under general anesthesia. Nalbuphine provided superior pain control, reduced the need for rescue analgesia, and resulted in higher patient satisfaction.

**Trial Registration:**

Trial registration at clinicaltrials.gov as NCT06594224.

## INTRODUCTION

Cesarean deliveries are currently the most frequent major surgical procedure performed globally. When planning for a cesarean section, the patient’s medical, surgical, and obstetric history, the resources, whether labor has started, and the urgency of the delivery should be clear to the anaesthetist.^1^,^2^ Neuraxial (epidural and spinal) procedures are the recommended technique for providing anaesthesia for Caesarean deliveries; the advantages and hazards of each technique determine the final decision. Although neuraxial procedures are usually favored when anaesthesia is applied during Caesarean delivery, there are absolute contraindications for spinal anesthesia, including patient refusal or uncooperating with the neuraxial method used, infection at the site of injection, severe aortic and mitral stenosis, and severe pulmonary hypertension.^3^,^4^

The best method of treating post-operative pain is by multimodal pharmacological and nonpharmacological means. The goal of multimodal analgesia techniques is to maximize the benefits of various analgesic modalities or pharmacological classes while limiting individual medications’ dosage and adverse effects withmultiple mechanisms of action. Medications that have been tried with variable degrees of success include different combinations of opioids, “nonsteroidal anti-inflammatory drugs (NSAIDs)”, acetaminophen, local anesthetics, and α2 agonists.^5^ NSAIDs block the synthesis of prostaglandins from arachidonic acid. NSAIDS are either selective, against cyclooxygenase 1 (cox 1), or nonselective, against cyclooxygenase 1 and cyclooxygenase 2 (cox 2). Ketorolac was the only available NSAID in IV form till the appearance of IV Ibuprofen.^6^,^7^

Several mechanisms are used to describe the way that ibuprofen produces its analgesic effect. Ibuprofen directly inhibits prostaglandin synthesis, neutrophil aggregation, and degranulation, as well as immune cell production of pro-inflammatory cytokines in vitro and in vivo. Also, ibuprofen’s analgesic effects may be related to elevated levels of the endocannabinoid anandamide.^8^-^10^ Nalbuphine which is a partial opioid antagonist having lesser respiratory depressant effect, better safety profile than other opioids, minimum circulatory effects, providing good sedation, and lower incidence of nausea and vomiting with significant analgesia.^11^ To the best of our knowledge, no study directly compares IV nalbuphine versus ibuprofen, so we aimed to evaluate the effectiveness of IV nalbuphine versus IV ibuprofen for postoperative pain relief following cesarean section under general anaesthesia.

## METHODOLOGY

This randomized controlled trial was conducted at the department of obstetrics & gynecology, Nishtar Hospital, Multan, Pakistan from March 2024 to August 2024.

### Ethical Approval:

It was obtained from “Institutional Ethical Committee” was taken (letter number: 12514/NMU, dated: August 7, 2024).

To detect a mean difference of two with population variance of 10, taking 95% confidence level and 80% post, the sample size was calculated to be 80 (40 in each group). The inclusion criteria were parturient females, aged between 18-45 years, gestational age above 32 weeks, BMI≤30 kg/m^2^, “American Society of Anesthesiologists (ASA)” Classification I to II, and planned to undergo elective cesarean. Patients were included only if they agreed to be a part of this research. Exclusion criteria were known allergy to the drugs being evaluated in this study. Women with renal and/or hepatic impairment (as per medical record), bronchial asthma (as per medical record, or platelets count <70000 (ul) were excluded. Women with bleeding diathesis, or history or risk of intracranial hemorrhage were also excluded. Informed and written consents were obtained.

At the time of enrollment, proper medical history was taken and detailed clinical examination done. Information like age, gestational age, BMI, duration of surgery, pulse rate, and blood pressure were noted. All women underwent general anesthesia and cesarean section employing standard protocols. After completion of the lower segment cesarean section, all parturients were shifted to ward to be put under observation. All women were randomly allocated to study groups. Females in Ibuprofen group were given 800 mg Ibuprofen in 200 ml normal saline IV (during 30 minutes) at the time of arrival in the ward. Women in Nalbuphine group received 20 mg nalbuphine in 200 ml normal saline IV (during 30 minutes) at the time of arrival in the ward.

**Fig.1:**
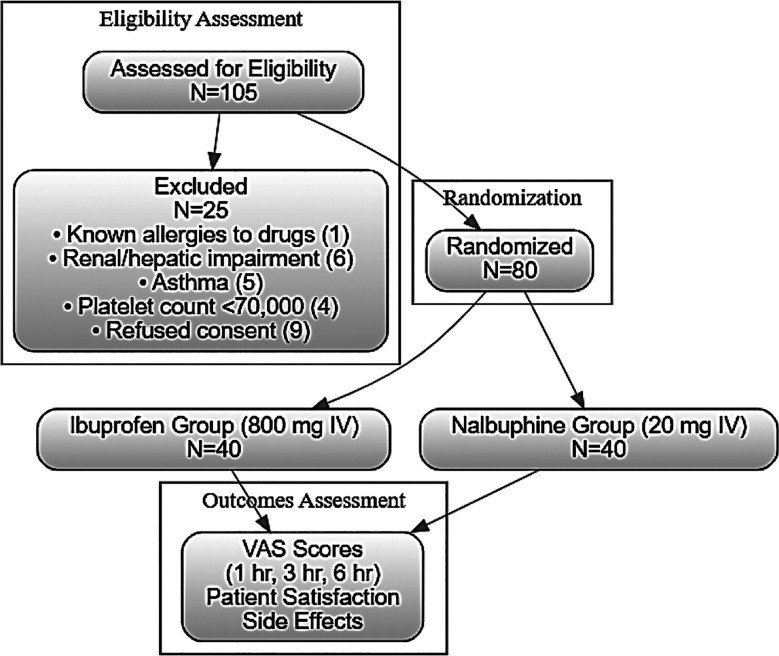
STROBE Flow Diagram.

All females were trained to use visual analog scale (VAS), presented as 10 cm horizontal line anchored by the words (translated in the local language) “no pain” (0) and “worst pain possible” (10). If VAS was ≥4, patients were administered rescue analgesia. Outcome was noted in the form of VAS pain scores post-surgery at one hour, three hours and six hours in both study groups. Patient’s satisfaction regarding their post-surgery management within six hours was noted on Likert scale taking score between one (not satisfied) to five (highly satisfied). Treatment related side effects like nausea, drowsiness, and gastrointestinal disturbances were noted. Fig.1 is showing STROBE flow diagram of this research.

Data were analyzed using “IBM-SPSS Statistics, version 26.0”. Qualitative data were shown as frequency and percentages. Quantitative variables were represented as mean and standard deviation. Independent sample t-test was used to compare numeric data, whereas chi-square test was employed to compare categorical data. P value < 0.05 was considered significant.

## RESULTS

The mean age was 32.10±8.67 years in the Ibuprofen group vs. 30.98±8.63 years in the Nalbuphine group (p=0.562). The BMI was 26.93±1.94 kg/m² in the Ibuprofen group and 26.61±1.98 kg/m² in the Nalbuphine group (p=0.479). Gestational age was 36.85±1.34 weeks in the Ibuprofen group vs. 36.50±2.29 weeks in the Nalbuphine group (p=0.500). In terms of ASA class, 77.5% of the Ibuprofen group and 85.0% of the Nalbuphine group were classified as ASA I (P = 0.390). The mean heart rate was 76.65±7.51 beats/min in the Ibuprofen group and 78.09±8.81 beats/min in the Nalbuphine group (P = 0.185). Oxygen saturation was 97.99±1.40% in the Ibuprofen group and 98.40±1.80% in the Nalbuphine group (P = 0.255). All baseline characteristics were statistically comparable between the two groups (Table-I).

**Table-I:** Comparison of Baseline characteristics of women in both study groups (N=80).

Characteristics		Ibuprofen group (n=40)	Nalbuphine group (n=40)	P-value
Age (years), Mean±SD		32.10±8.67	30.98±8.63	0.562
Body mass index (kg/m2), Mean±SD		26.93±1.94	26.61±1.98	0.479
Gestational age (weeks), Mean±SD		36.85±1.34	36.50±2.29	0.500
American Society of Anesthesiologists class	I	31 (77.5%)	34 (85.0%)	0.390
	II	9 (22.5%)	6 (15.0%)
Systolic blood pressure (mm Hg), Mean±SD		119.52±16.96	121.36±15.10	0.610
Diastolic blood pressure (mm Hg), Mean±SD		80.17±10.43	78.48±9.12	0.443
Heart rate (beats/min), Mean±SD		76.65±7.51	78.09±8.81	0.185
Oxygen saturation (%)		97.99±1.40	98.40±1.80	0.255

**Fig.2:**
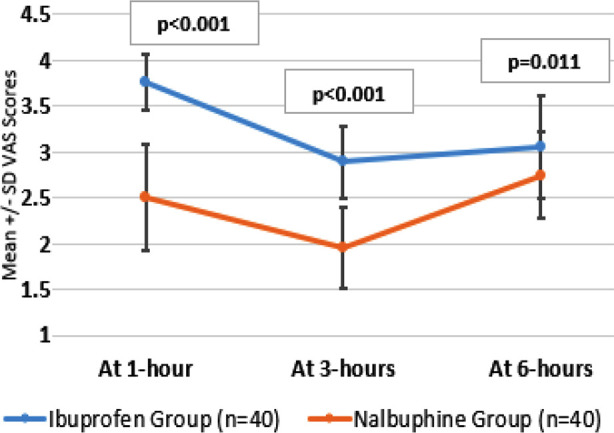
Comparison of post-surgery VAS scores in both study groups (N=80).

Post-surgery, nalbuphine group demonstrated better pain control in the postoperative period compared to Ibuprofen, at 1-hour (p<0.001), at 3-hours (p<0.001), and at 6-hours (p=0.011), as shown in Fig.2.

In the comparison of post-surgery outcomes between the Ibuprofen and Nalbuphine groups (Table-II), rescue analgesia was significantly more common in the Ibuprofen group, with 28 patients (70.0%) requiring it, compared to only seven patients (17.5%) in the Nalbuphine group (p<0.001). Regarding patient satisfaction, 25 patients (62.5%) in the Nalbuphine group reported being highly satisfied, while in the Ibuprofen group, only five patients (12.5%) were highly satisfied (p<0.001). For nausea, eight patients (20.0%) in the Nalbuphine group experienced this side effect, compared to four patients (10.0%) in the Ibuprofen group (p=0.210). Drowsiness was significantly more frequent in the Nalbuphine group, affecting 10 patients (25.0%), compared to two patients (5.0%) in the Ibuprofen group (p=0.012). Gastrointestinal discomfort was observed in five patients (12.5%) in the Ibuprofen group, while only one patient (2.5%) in the Nalbuphine group reported this side effect (p=0.090).

**Table-II:** Comparison of post-surgery outcomes (N=80).

Outcomes		Ibuprofen group (n=40)	Nalbuphine group (n=40)	P-value
Rescue analgesia		28 (70.0%)	7 (17.5%)	<0.001
Satisfaction	Not sure	25 (62.5%)	7 (17.5%)	<0.001
	Satisfied	10 (25.0%)	15 (37.5%)	
	Highly satisfied	5 (12.5%)	18 (45.0%)
Nausea		4 (10.0%)	8 (20.0%)	0.210
Drowsiness		2 (5.0%)	10 (25.0%)	0.012
Gastrointestinal discomfort		5 (12.5%)	1 (2.5%)	0.090

## DISCUSSION

Postoperative pain management is crucial in cesarean deliveries, as inadequate pain control may impair recovery and lead to delayed ambulation, prolonged hospital stay, and potential long-term complications.^12^,^13^ Effective analgesia also facilitates early breastfeeding and bonding with the newborn, which are key aspects of post-cesarean recovery.^14^ The findings of this study suggested that Nalbuphine provided superior pain relief in the immediate postoperative period compared to Ibuprofen. The VAS scores were significantly lower in the Nalbuphine group at one hour (p<0.001), three hours (p<0.001), and six hours (p=0.011) post-operatively. Raju et al., also found that Nalbuphine was more efficacious than paracetamol in controlling postoperative pain following cesarean sections.^11^ Their study revealed that patients receiving Nalbuphine had significantly lower numeric rating scores for pain, and required fewer doses of rescue analgesics compared to patients receiving paracetamol. Raju et al. also reported that the number of rescue analgesic doses was significantly lower with Nalbuphine (p<0.001).^11^ These findings highlight the effectiveness of Nalbuphine in reducing the overall opioid consumption postoperatively, which is a crucial consideration in minimizing opioid-related side effects and preventing opioid misuse.

Liu et al.^15^ demonstrated that Nalbuphine combined with sufentanil provided better postoperative pain control than sufentanil alone, with patients in the Nalbuphine group reporting lower VAS scores and higher satisfaction levels. In contrast, our study focused on the comparison with Ibuprofen, an NSAID, which has a different mechanism of action than paracetamol or opioids like sufentanil, making our study’s contribution to the literature on pain management after cesarean sections more relevant for clinical settings where NSAIDs are commonly used. The analgesic superiority of Nalbuphine over Ibuprofen can be explained by the opioid’s ability to act as a Kappa-opioid receptor agonist and μ-opioid receptor antagonist, which contributes to its dual mechanism of providing analgesia while reducing some of the side effects associated with pure μ-opioid agonists.^16^ In contrast, Ibuprofen works by inhibiting cyclooxygenase (COX) enzymes, thereby reducing the production of prostaglandins, which mediate pain and inflammation.^17^

In the present study, the need for rescue analgesia was significantly higher in the Ibuprofen group (70%) compared to the Nalbuphine group (17.5%) (p<0.001). This result mirrors the findings of Sun et al.^18^, who found that patients receiving Nalbuphine for post-cesarean pain required fewer additional doses of analgesia compared to those receiving sufentanil.

Our study found a significant difference in patient satisfaction between the two groups, with 62.5% of patients in the Nalbuphine group reporting highly satisfied compared to only 12.5% in the Ibuprofen group (p < 0.001). Liu et al. also reported higher patient satisfaction in the Nalbuphine group, with satisfaction levels being significantly better at 6 and 12 hours postoperatively.^15^ The higher satisfaction rates in our study are likely due to the superior pain control achieved with Nalbuphine, as effective pain management is closely linked to patient satisfaction in the postoperative period. Ismail et al.^19^ found that patients who received patient-controlled analgesia (PCA) with pethidine were more satisfied with their pain management than those who received continuous opioid infusion. While our study did not utilize PCA, the concept of patient-centered pain management and reducing breakthrough pain with effective analgesia aligns with these findings.

In terms of side effects, we observed that 20% of patients in the Nalbuphine group experienced nausea compared to 10% in the Ibuprofen group (p=0.210). It is worth noting that opioid-based analgesics like Nalbuphine are generally associated with a higher incidence of nausea and vomiting due to their effects on the central nervous system. Liu et al.^15^ found similar results, with fewer patients in the Nalbuphine group experiencing nausea and vomiting compared to the sufentanil group (p = 0.008). Sedation is a well-documented side effect of opioids, including Nalbuphine, and while it may not always be harmful, it should be considered in the context of patient safety and recovery.^20^ Despite the higher incidence of drowsiness, Nalbuphine’s overall effectiveness in pain relief and patient satisfaction may outweigh this drawback, especially in controlled hospital environments where patients can be closely monitored. Gastrointestinal discomfort was noted in 12.5% of patients in the Ibuprofen group and 2.5% in the Nalbuphine group (p=0.090). These are well-known gastrointestinal side effects of NSAIDs like Ibuprofen, which can cause irritation of the gastric lining and increase the risk of gastrointestinal bleeding. Shehab et al.^21^ compared IV Ibuprofen with Ketorolac and similarly found that patients in the Ibuprofen group had lower VAS scores and fewer side effects, including gastrointestinal issues. While NSAIDs like Ibuprofen can be effective for postoperative pain relief, these might carry a risk of gastrointestinal disturbances, especially with higher doses or prolonged use.

Although, we did not evaluate hemodynamic parameters post-delivery among studied women but researchers in the past have suggested that Nalbuphine provided better hemodynamic stability compared to paracetamol (p<0.05). Sun et al.,^18^ and Liu J et al.^15^ found no significant hemodynamic instability in patients receiving Nalbuphine. The consistency in findings across these studies suggests that Nalbuphine, when administered at appropriate doses, does not compromise cardiovascular stability, making it a safe option for postoperative pain management in cesarean sections.

While our study focused on comparing Nalbuphine to Ibuprofen, the consistent theme across studies is Nalbuphine’s superior pain relief and reduced need for additional analgesics, without significantly increasing the incidence of side effects. This makes Nalbuphine a highly effective option for post-cesarean pain management, particularly when opioids are warranted but there is a desire to minimize the risks associated with pure μ-opioid agonists.

### Limitations:

Relatively small sample size might have affected the generalizability of this study. The short follow-up period of only six hours post-surgery limits the assessment of long-term pain relief and potential late-onset side effects. Patient-reported outcomes such as satisfaction may be subject to bias, as they are influenced by individual expectations and subjective experiences of pain relief.

## CONCLUSION

This study demonstrated that IV Nalbuphine was more effective than IV Ibuprofen for postoperative pain relief following cesarean section under general anesthesia. Nalbuphine provided superior pain control, reduced the need for rescue analgesia, and resulted in higher patient satisfaction.
